# Rehabilitation care workers address environmental factors with persons with disabilities

**DOI:** 10.4102/ajod.v14i0.1609

**Published:** 2025-05-26

**Authors:** Judith N. Mahlangu, Theresa Lorenzo, Eve M. Duncan

**Affiliations:** 1 Department of Family, Community and Emergency Care, Faculty of Health Sciences, University of Cape Town, Cape Town, South Africa; 2 Department of Health and Rehabilitation Sciences, Faculty of Health Sciences, University of Cape Town, Cape Town, South Africa

## Introduction

Our opinion article stems from a research project that investigated the contribution of rehabilitation care workers (RCWs), also called community rehabilitation workers (CRWs), to strengthening rehabilitation in community-based services at a primary level of care in two peri-urban districts. Rehabilitation care workers are a human resource support system at community level that addresses the needs of persons with disabilities in equalising opportunities for social and economic development (Ned et al. 2020; Philpott, McLaren & Rule 2020). They work across health, education, livelihoods, and social public service sectors to strengthen the own empowerment of persons with disability in accordance with the World Health Organization (WHO) Community-based Rehabilitation (CBR) Matrix and CBR Guidelines (WHO 2010).

Rehabilitation care workers in the two research sites provide support to persons with disabling impairments discharged from a hospital and/or a transitional level of care facility in the government provincial health system by following up on individualised rehabilitation plans under supervision of a rehabilitation professional. They take the prevailing social determinants of health into account and where possible address the environmental factors that affect the functional recovery, psychological well-being, social participation, and equitable inclusion of persons with disability (Frier et al. 2018; Lippi et al. 2022).

The International Classification of Functioning, Disability and Health (ICF) (WHO 2001) and the CBR Guidelines (WHO 2010) are useful conceptual tools for identifying and addressing environmental factors influencing the community participation and social inclusion of persons with disability (Rhoda et al. 2016). The ICF identifies five categories of environmental factors: products and technology; natural and built environment; support and relationships; attitudes, and lastly, policies, systems and services. Each of the five components of the CBR Guidelines (health, education, livelihood, social and empowerment) consists of five elements, all of which warrant consideration by RCWs to promote the provision of comprehensive, multi-sectoral support for persons with disabilities and their families. The purpose of our opinion article is to highlight some of the ways in which environmental factors in the two districts affected the ability of RCWs to deliver services to persons with disability.

## Background to rehabilitation care workers

In 2012, the Western Cape provincial health department and the University of Cape Town launched an initiative that aimed at developing a suitably trained mid-level disability inclusive health workforce in the primary healthcare (PHC) system. A 1-year Higher Certificate in Disability Practice (HCDP) was designed by a team of rehabilitation professionals and disability practitioners and registered with the South African Qualification Authority at Level 5 of the National Qualification Framework. The first cohort of 28 students graduated in 2014. A total of 120 RCWs have graduated to date, most of whom are employed in the peri-urban government posts or by non-profit organisations (NPOs) with their salaries paid by the Department of Health. Some work in collaboration with rehabilitation professionals as members of a rehabilitation community services team that is being piloted in the two metropole districts. We anticipate that the findings will inform disability service planners and human resource managers in other CBR service settings about the job expectations and work conditions of RCWs.

## Research project on which this opinion article is based

A collaborative inquiry was conducted with members of the community services rehabilitation teams in the two pilot districts with the aim of describing the contribution of a disability inclusive health workforce to the PHC system. This opinion article is based on one of the objectives, which was to map the type and range of services rendered by HCDP trained RCWs and to highlight how environmental factors in the two districts affected the ability of RCWs to deliver services to persons with disabilities.

Purposefully sampled participants included eight HCDP alumni RCWs; eight persons with disabilities that received their services, and three occupational therapists, one speech therapist, and one social worker who supervised the RCWs. Data were gathered with informed consent during three workshops of 4-h duration. Data were presented in a Venn diagram of intersectoral services followed by a tape-recorded discussion of barriers and facilitators to service delivery and a retrospective checklist of RCW competences based on the HCDP curriculum outcomes followed by a tape-recorded discussion of perceived strengths and gaps in RCW training. Our opinion article is based on the audio data of discussions among the eight HCDP alumni RCWs. The audio data were transcribed into textual data and deductively coded using the five ICF environmental factors as categories. Appropriate ethics approval from the relevant organisations was obtained.

## Findings and reflections

Findings are presented under each of the ICF environmental categories and substantiated with quotes from the data in italics.

### Category 1: Providing assistive products and technology

The time delay between application for and delivery of an assistive product and the associated financial costs made it difficult for RCWs to help persons with disability and their families:

‘Also with assistive devices, wheelchairs and walking frame, patients can make applications, it can take them six months to wait for assistive device. Persons with disabilities want to purchase the wheelchair, but they can’t afford it.’ (RCW, K, Female)

Assistive products and technology (AP and AT) services are hampered by barriers in procurement and delivery systems, inadequate integration of AP and AT services across service providers, inadequate AP and AT knowledge among service providers and insufficient numbers of service providers (Visagie et al. 2020). Rehabilitation care workers trained in the basics of AP and AT can help bridge some of these barriers. A systems approach by health system planners at a primary level of care is advocated to facilitate seamless, equitable AP and AT service delivery (Visagie et al. 2020).

### Category 2: Making the natural and built environment accessible

Rehabilitation care workers identified features of the natural environment (e.g. climate, terrain) and built environment (e.g. human-made changes) that limited the participation of persons with disabilities:

‘We talking about our parks and beaches as well, some of our patients would want to go but … there are no ramps … we have a person with disability and they unable to access that specific area in their home.’ (RCW, K, Female)

The social environment in the two research sites also curtailed the ability of RCWs to assist clients:

‘Unemployment, that leads to poverty, crime, gangs and drugs and dependency on grants.’ (RCW, M, Female)

Precarious environmental safety concerns and adverse social determinants of health affect the productivity and well-being of persons with disabilities (Frier et al. 2018). Rehabilitation care workers working in complex environments benefit from stakeholder support, a multidisciplinary approach and positive guidance provided by rehabilitation professionals (Harriparsad & Dlungwane 2022).

### Category 3: Creating supportive relationships

Rehabilitation care workers highlighted the significance of their role in facilitating supportive family relationships and that the relational complexities of this role contributed to work overload and stress:

‘Family support is a very, very big thing that we [*RCWs*] focus on in the community and homes. Sometimes there is a very good positive spouse support as well as family support, but when it comes to another homes there is absolutely no support … which results in us overworking ourselves.’ (RCW, K, Female)

Systematic reviews suggest that organisational as well as individual interventions may help to reduce burnout and build resilience in health workers that are exposed to significant occupational stress (Thom 2020). Self-empowerment of mid-level health workers is indicated through widening their perspectives on participating in continuing education self-management programmes (Johnson et al. 2022).

### Category 4: Addressing stigmatising attitudes

Rehabilitation care workers reported that the HCDP equipped them to discern and address stigmatising attitudes towards persons with disabilities. They also faced resistant attitudes and limited recognition of their role:

‘We also talking about attitudes of our health professionals. Sometimes they don’t show any empathy…. They talk about a person without a person.’ (RCW, K, Female)

Rehabilitation care workers can be effectively utilised in CBR if their role is understood and their potential is not limited by professional protectionism and scepticism (Philpott et al. 2020). Differentiating the RCW scope of practice and how to collaborate with this cadre through task sharing and task shifting will overcome resistance by health professionals (Gamiet & Rowe 2019).

### Category 5: Making services, systems and policies more inclusive

Rehabilitation care workers knew how public, non-governmental and other sectors operated in the two geographic study locations and were familiar with the structural framework for disability inclusion. They reported that services, systems and policies created multiple bureaucratic and access barriers:

‘As much as services are available in communities, they are not easily accessible for persons with disabilities … they experience a lot of red tape. Transport is available; however, it is not affordable. The waiting for appointments is long with both consultation and collecting of medications. Also inappropriate referrals to us from hospitals.’ (RCW, M, Female)

Acknowledging the grassroots contributions of RCWs to inclusive development requires a re-evaluation of existing hierarchies in health systems and health education that place physicians and curative services at the highest level of influence and authority (Mahlangu et al. 2024).

## Putting it all together

The RCWs in our study lived and worked in the communities that they served. Although the HCDP equipped them to support the rehabilitation, participation and social inclusion of the persons with disabilities, their contribution to health system strengthening was constrained by environmental factors. Our findings highlight the need for health workforce education and training to include strategies for dealing with environmental barriers and social determinants of health (WHO 2023). More needs to be done to recognise the role that RCWs play in the rehabilitation discipline by addressing their work conditions and supporting their employee well-being. Doing so will promote the WHO Rehabilitation 2030 Call for Action to prioritise rehabilitation within health systems (Philpott et al. 2020).
